# Accelerated Reaction Rates within Self-Assembled Polymer Nanoreactors with Tunable Hydrophobic Microenvironments

**DOI:** 10.3390/polym12081774

**Published:** 2020-08-07

**Authors:** Andrew Harrison, Michael P. Zeevi, Christopher L. Vasey, Matthew D. Nguyen, Christina Tang

**Affiliations:** College of Chemical and Life Sciences Engineering, Virginia Commonwealth University, Richmond, VA 23284-3028, USA; harrisona3@vcu.edu (A.H.); zeevimp@vcu.edu (M.P.Z.); vaseycl@vcu.edu (C.L.V.) nguyenmd4@vcu.edu (M.D.N.)

**Keywords:** self-assembly, nanoreactor, micelle, amphiphilic, nanoparticle, hybrid, nanoprecipitation, solubility parameters

## Abstract

Performing reactions in the presence of self-assembled hierarchical structures of amphiphilic macromolecules can accelerate reactions while using water as the bulk solvent due to the hydrophobic effect. We leveraged non-covalent interactions to self-assemble filled-polymer micelle nanoreactors (NR) incorporating gold nanoparticle catalysts into various amphiphilic polymer nanostructures with comparable hydrodynamic nanoreactor size and gold concentration in the nanoreactor dispersion. We systematically studied the effect of the hydrophobic co-precipitant on self-assembly and catalytic performance. We observed that co-precipitants that interact with gold are beneficial for improving incorporation efficiency of the gold nanoparticles into the nanocomposite nanoreactor during self-assembly but decrease catalytic performance. Hierarchical assemblies with co-precipitants that leverage noncovalent interactions could enhance catalytic performance. For the co-precipitants that do not interact strongly with gold, the catalytic performance was strongly affected by the hydrophobic microenvironment of the co-precipitant. Specifically, the apparent reaction rate per surface area using castor oil (CO) was over 8-fold greater than polystyrene (750 g/mol, PS 750); the turnover frequency was higher than previously reported self-assembled polymer systems. The increase in apparent catalytic performance could be attributed to differences in reactant solubility rather than differences in mass transfer or intrinsic kinetics; higher reactant solubility enhances apparent reaction rates. Full conversion of 4-nitrophenol was achieved within three minutes for at least 10 sequential reactions demonstrating that the nanoreactors could be used for multiple reactions.

## 1. Introduction

Supramolecular assemblies such as those achieved through self-assembly of amphiphilic molecules are a promising alterative to organic solvent for performing chemical reactions. The reaction occurs in the hydrophobic compartment of the self-assembled “nanoreactor” structure. These amphiphilic structures can be dispersed in water, which allows reactions traditionally performed in an organic solvent to be carried out in a bulk aqueous phase. Replacing an organic solvent with these “nanoreactors” dispersed in water may reduce solvent waste. Furthermore, when the reaction occurs in the hydrophobic compartment of the self-assembled nanoreactor structure, the reactions is confined to a small volume which can improve efficiency and selectivity of the reaction compared to the same reaction done on a larger volume scale [1,2,3].

Supramolecular assemblies of small molecule amphiphiles have been used to perform a wide range of reactions. For example, Lipshutz and co-workers have developed a series of “designer surfactants” for micellar catalysis carried out in water using several generations of amphiphilic surfactants with polyethylene glycol hydrophilic blocks linked to various lipids. These surfactants have been used as alternative solvents for a wide range of organic reactions such as cross-couplings [4,5,6], oxidations [7], reductions [8] and peptide synthesis [9] with high yield. Water was used as the bulk solvent; the reaction occurred in the hydrophobic compartment created by dynamic self-assembly of the amphiphilic molecules. This dynamic self-assembly was driven by non-covalent interactions such as hydrophobic interactions of the lipid portions of the amphiphilic surfactants. The surfactant and resulting supramolecular assembly affected the catalytic performance. For example, by designing a surfactant molecule with longer PEG portion and a shorter carbon linker between vitamin E and PEG, the micelles were larger and faster reactions were observed [10] in various transition metal-catalyzed reactions (e.g., Suzuki–Miyaura, cross metathesis, amination, C–H activation, borylation, silylation, etc.). Changing the hydrophobic portion of the surfactant from vitamin E to β-sitosterol improved conversion of cross metathesis reactions [10,11]. Surfactants with polar sulfone components within the nonpolar, hydrophobic cores hava recently been reported which facilitate peptide synthesis [9]. It is important to note design of these systems has generally involved syntheses of libraries of new amphiphilic molecules and screening their reactivity [9].

Self-assembled macromolecular amphiphilic nanoreactor systems have also been considered. Such systems have improved stability compared to self-assembled small molecule amphiphilic systems [12,13]. For example, O’Reilly and co-workers incorporated 4-(*N*,*N*-dimethylamino)pyridine, a nucleophilic catalyst for esterification reactions, into the hydrophobic block of an amphiphilic block copolymer using reversible addition–fragmentation chain (RAFT) polymerization. The resulting polymer self-assembled into kinetically frozen micelles. The stable, micellar nanoreactors catalyzed the competitive esterification between multiple anhydrides. Higher conversions were observed with more hydrophobic substrates. Further, the hydrophobicity of the substrate could also be used to modify the selectivity [12,13]. In other work, L-proline, a chiral organocatalyst for the aldol reaction, was incorporated into the hydrophobic block of an amphiphilic block copolymer via RAFT polymerization. Upon self-assembly in water, core-shell micellar nanoreactors with catalytic hydrophobic cores were achieved. The nanoreactors were significantly more efficient than the unsupported catalyst [14], which was attributed to the hydrophobic microenvironment of the micelle core. The catalyst loading and core hydrophobicity affected the turnover number, but the effects could not be decoupled from micelle swelling.

Hierarchical assemblies of metal nanoparticle catalysts and self-assembled polymers have also been used as effective nanoreactors. For example, micellar nanoreactors of gold nanoparticles stabilized by water soluble 1, 2, 3-triazlyl dendronized polymers have also been reported by Liu et al. The dendronized polymer–gold nanoparticle composite formed self-assembled micelles in water and was used to catalyze the reduction of 4-nitrophenol to 4-aminophenol. They observed that the catalytic performance was affected by the length and architecture (i.e., linear or branched) of the polymer stabilizer. Turnover frequencies (TOF) were reported to be as high as 7350 h^−1^. These results demonstrate that properties of the self-assembled polymer microenvironment influence the catalytic performance. Varying the properties of the polymer microenvironment in this case requires synthesis of different types of 1, 2, 3-triazlyl ligands as well as click-compatible polymer tails [15].

Systematic investigations to understand the effect of the hydrophobic microenvironment core material on the nanoreactor performance with respect to catalyst performance have yet to be established. Such studies of the effect of nanoreactor composition on the catalytic performance are limited. With micellar (small molecule and macromolecular) nanoreactors, such a study necessitates synthesis of a new amphiphilic stabilizer for each nanoreactor.

As an alternative to systems that require unique synthesis of amphiphilic molecules to vary the properties of the hydrophobic compartment, flash nanoprecipitation offers a simple approach to produce filled polymer micellar nanoreactors with different core materials. The hydrodynamic size and catalyst concentration in the nanoreactor dispersion can be controlled in order to study the effects of composition of reactor performance. Therefore, in this work, we formulate self-assembled filled polymer micelle nanoreactors encapsulating gold nanoparticle catalysts with various hydrophobic microenvironments. We study the effect of the hydrophobic co-precipitants on self-assembly by analyzing the resulting hydrodynamic size via dynamic light scattering and incorporation efficiency of the gold nanoparticles via ICP-OES. We evaluate the catalytic activity using reduction of 4-nitrophenol (4NP) with sodium borohydride as a model reaction and compare turnover frequencies to previously reported gold–polymer hybrid nanoparticle systems. Intrinsic kinetics evaluated using the Langmuir–Hinshelwood model and experimentally measured partition coefficients to estimate the local reactant concentration in the reactor are discussed. Nanoreactor stability when performing sequential reactions is also investigated.

## 2. Materials and Methods

### 2.1. Materials

Polystyrene (PS, *M*_w_ 750 g/mol) was purchased from Polymer Source, Inc (Dorval, QC, Canada). Sodium borohydride, 4-nitrophenol, dodecane, dodecanethiol and potassium chloride were purchased from Sigma Aldrich (St. Louis, MO, USA). Castor oil was purchased from Alfa Aesar (Haverhill, MA, USA). Dodecylamine was purchased from Beantown Chemical Corporation (Hudson, NH, USA). Dodecanethiol (DDT)-stabilized 5-nm nanoparticles, tetrahydrofuran (tetrahydrofuran (THF), HPLC grade), ethanol (ACS reagent grade) and diethyl ether (ACS reagent grade) were purchased from Fisher Scientific (Fairmont, NJ, USA). Aqueous dispersions of 5 nm PEG-coated gold nanoparticles were obtained from Nanocs (New York, NY, USA); aqueous dispersions of 5-nm citrate-stabilized gold nanoparticles were purchased from Ted Pella (Redding, CA, USA). Environmental Grade Hydrochloric Acid 30%–38% and Environmental Grade Nitric Acid 70% were purchased from GFS Chemicals (Columbus, OH, USA). The ^1^H-NMR solvent D_2_O with 4,4-dimethyl-4-silapentane-1-sulfonic acid DSS as an internal standard was purchased from Cambridge Isotope Lab, Inc (Andover, MA, USA). These chemicals and materials were used as received. Polystyrene–*b*–polyethylene glycol (PS–*b*–PEG, PS_m_–*b*–PEG_n_ where m = 1600 g/mol and n = 5000 g/mol) was obtained from Polymer Source (Product No. P13141-SEO). Prior to use, PS–*b*–PEG was dissolved in THF (500 mg/mL) and precipitated in ether (~1:20 *v/v* THF:ether). The PS–*b*–PEG was recovered by centrifuging, decanting and drying under vacuum at room temperature for 2 days as previously described [16].

### 2.2. Nanoreactor Assembly

Initially, the as-received dodecanethiol-stabilized gold nanoparticles in toluene (1 mL) were precipitated in ethanol (45 mL) and filtered using a Buchner funnel. The filtered nanoparticles were resuspended in THF and concentrated via evaporation at room temperature overnight to achieve a nominal concentration of 20 mg/mL for nanoreactor self-assembly. The final concentration was confirmed by inductively coupled plasma optical emission spectroscopy using an Agilent 5110 (ICP-OES, Santa Clara, CA, USA). UV spectra collected on an Ocean Optics FLAME-S-UV-vis with a HL-2000-FHSA light source (Largo, FL, USA) were compared before and after the solvent switch to confirm processing did not significantly affect gold nanoparticle size.

Polymer nanoreactors were self-assembled via flash nanoprecipitation similar to previous reports [16]. Briefly, PS–*b*–PEG (12 mg), dodecanethiol-stabilized 5-nm gold nanoparticles (1 mg) and castor oil co-precipitate, abbreviated CO (11 mg) were added to 1 mL of tetrahydrofuran (THF) and sonicated at 55 °C for 30 min. Using a manually operated confined impinging jet mixer with dilution (CIJ-D) [17,18] the resulting THF mixture was rapidly mixed against 1 mL of water. Reynolds numbers >1300 were achieved. The effluent of the mixer was immediately diluted with 8 mL of water. The resulting dispersion (10 mL total) of nanoreactors was stored at room temperature for further characterization and analysis without purification.

For comparison, we formulated nanoreactors incorporating gold nanoparticle catalysts with various hydrophobic microenvironments. To create nanoreactors with various microenvironments, a different co-precipitate was used in place of castor oil. The various microenvironments used in this study were dodecane, dodecylamine, dodecanethiol and polystyrene (molecular weight 750 g/mol PS 750).

We were also interested in evaluating the effect of the amphiphilic nature of the self-assembled nanoreactor. Thus, we compared the catalytic performance to PEG-coated gold nanoparticles as well as gold nanoparticles added to preformed polymer nanoparticles. For comparison to nanoreactors prepared via flash nanoprecipitation, we added gold nanoparticles to preformed castor oil nanoparticles (CO NP w AuNP). Following flash nanoprecipitation, gold nanoparticles suspended in THF (500 µL, 2 mg/mL) was added dropwise to a stirring a 5-mL dispersion of preformed castor oil nanoparticles over 5 min.

### 2.3. Nanoreactor Characterization

Nanoreactor size (i.e., hydrodynamic diameter) was measured after mixing using a Malvern Zetasizer Nano ZS (Westborough, MA, USA) with a backscatter detection angle of 173°. Intensity weighted size distributions are reported using the average of four measurements of the intensity weight distributed with normal resolution. The reported size is the peak 1 mean intensity. The polydispersity index (PDI) is defined from the moment of the cumulant fit of the autocorrelation function calculated by the instrument software (appropriate for samples with PDI <0.3) and is reported as a measure of particle size distribution [19]. For stability analysis, reaction solutions were allowed to sit for at least 24 h prior to analysis to reduce the formation of bubbles within the solution.

UV absorbance spectra (300 to 1200 nm) of the nanoparticle dispersions were measured at room temperature with an Ocean Optics FLAME-S-UV-vis with a HL-2000-FHSA light source (Largo, FL, USA) after formulation.

For visualization by TEM, samples were prepared by submerging a grid in a diluted nanoreactor solution (1:10) for 1 h. After submersion, the grids were removed from the solution and dried at ambient conditions overnight. Samples were imaged using a Zeiss Libra 120 TEM (Oberkochen, Germany) using an accelerating voltage of 120 kV.

To determine the gold concentration in the nanoreactor dispersions, the nanoreactor dispersions were dissolved in THF and digested in aqua regia (1:3 nitric acid:hydrochloric acid by volume) for at least 24 h. The samples were then diluted to 5% *v/v* aqua regia. Gold concentration of the digested sample was measured using inductively coupled plasma optical emission spectroscopy measurements with an Agilent 5110 (Santa Clara, CA, USA). A matrix modifier, potassium chloride (2 mg/mL) in 5% *v/v* aqua regia, was used to increase the ion concentration to improve the peak resolution. The reported concentrations are the average of three trials.

To determine the incorporation efficiency of the catalytic gold nanoparticles into the nanocomposite nanoreactor structure during self-assembly, the nanoreactor dispersions were extracted with an equal volume of diethyl ether thrice to remove gold nanoparticles that were unassociated with the nanoreactors. The gold content in the aqueous phases following extraction were analyzed by ICP–OES. Incorporation efficiency was calculated according as the fraction of gold associated with the nanoreactor after extraction with ether compared to the total gold concentration in the dispersion.

### 2.4. Kinetic Analysis

The catalytic performance of the nanoreactors was evaluated using the reduction of 4-nitrophenol with sodium borohydride as a model reaction using well established procedures [20,21]. Briefly, to the nanoreactors (0.0079 mol % AuNP) were added 4-nitrophenol (20 µL, 0.01 M, abbreviated 4NP) followed by aqueous sodium borohydride (within 5 min of preparation, abbreviated NaBH_4_) to form a 2-mL reaction solution. The final reaction mixture contained less than 0.01 vol % THF that would have been residual from the self-assembly process. The reduction of 4-nitrophenol was monitored using UV spectroscopy (Ocean Optics FLAME-S-VIS-NIR-ES, Largo, FL, USA, with a HL-2000-FHSA light source (300–1200 nm) with a CUV-UV cuvette holder placed on a stir plate. The induction time and apparent reaction rate (*K*_app_) were determined from tracking the absorbance at 425 nm as a function of time. The values of *K*_app_ and induction time are the averages (± standard deviations) of at least 3 trials of each experiment. Detailed procedures are provided in the Supporting Information. To vary the sodium borohydride concentration, the sodium borohydride solution had an initial concentration of 6 M and the nanoreactor solution and sodium borohydride addition solution volumes were adjusted such that the final reaction volume was 2 mL.

### 2.5. Partition Coefficient Determination

To better understand the reactant concentration in the hydrophobic microenvironment of the nanoreactor, our goal was to determine the partition coefficient of the 4-nitrophenol between water and the castor oil which is a ratio of the concentration in the hydrophobic microenvironment to the bulk aqueous phase. To estimate this partition coefficient, an aqueous solution of 4-nitrophenol (0.1-mM) was placed in equilibrium with castor oil. For comparison to polystyrene nanoreactor, toluene was used as a proxy for polystyrene. After vigorous shaking for 10 min, the emulsion was allowed to rest for 1 week prior to analysis. After 1 week, UV-vis analysis was used to analyze the absorbance of 4-nitrophenol in the original solution and the aqueous phase of two phase equilibrium. According to Beer’s law (concentration is proportional to absorbance), the partition coefficient was calculated using the absorbance at 425 nm:

(1) $$P_{4\mathrm{NP}}=\frac{{\left[4NP\right]}_{\mathrm{hydrophobic}}}{{\left[4NP\right]}_{\mathrm{aq}}}=\frac{1-\frac{Abs_{4{\mathrm{NP}}_{\mathrm{aq}}}}{Abs_{4{\mathrm{NP}}_{i}}}}{Abs_{4{\mathrm{NP}}_{\mathrm{aq}}}}$$

where $P_{4\mathrm{NP}}$ is the partition coefficient is the ratio of the of 4-nitrophenol in the hydrophobic microenvironment of the nanoreactor core and water, $Abs_{4{\mathrm{NP}}_{\mathrm{aq}}}$ is the absorbance of 4NP in the aqueous phase of the equilibrium and $Abs_{4{\mathrm{NP}}_{i}}$ is the absorbance of 4NP in the initial solution of water prior to being placed into equilibrium with the hydrophobic phase. The absorbances were averages of three measurements.

### 2.6. Langmuir–Hinshelwood Kinetics

For more detailed kinetic analysis, we performed full kinetic analysis considering the two-step reaction mechanism previously established [21]. Importantly, to consider the effect of localized reagent concentrations, the partition coefficient for 4-nitrophenol was incorporated and the referenced Langmuir–Hinshelwood mechanistic equations were adapted as follows:

(2) $$-\left(\frac{dc_{\mathrm{nip}}}{dt}\right)=k_{a}S\frac{{\left(K_{\mathrm{nip}}P_{\mathrm{nip}}c_{\mathrm{nip}}\right)}^{n}\left(K_{{\mathrm{BH}}_{4}}c_{{\mathrm{BH}}_{4}}\right)}{{\left[1+{\left(K_{\mathrm{nip}}P_{\mathrm{nip}}c_{\mathrm{nip}}\right)}^{n}+K_{\mathrm{Hx}}c_{\mathrm{Hx}}+K_{{\mathrm{BH}}_{4}}c_{{\mathrm{BH}}_{4}}\right]}^{2}}$$

(3) $$\left(\frac{dc_{Hx}}{dt}\right)=k_{a}S\frac{{\left(K_{\mathrm{nip}}P_{\mathrm{nip}}c_{\mathrm{nip}}\right)}^{n}\left(K_{{\mathrm{BH}}_{4}}c_{{\mathrm{BH}}_{4}}\right)}{{\left[1+{\left(K_{\mathrm{nip}}P_{\mathrm{nip}}c_{\mathrm{nip}}\right)}^{n}+K_{\mathrm{Hx}}c_{\mathrm{Hx}}+K_{{\mathrm{BH}}_{4}}c_{{\mathrm{BH}}_{4}}\right]}^{2}}\;-k_{b}S\frac{K_{\mathrm{Hx}}c_{\mathrm{Hx}}K_{{\mathrm{BH}}_{4}}c_{{\mathrm{BH}}_{4}}}{{\left[1+{\left(K_{\mathrm{nip}}P_{\mathrm{nip}}c_{\mathrm{nip}}\right)}^{n}+K_{\mathrm{Hx}}c_{\mathrm{Hx}}+K_{{\mathrm{BH}}_{4}}c_{{\mathrm{BH}}_{4}}\right]}^{2}}$$

where $P_{\mathrm{nip}}$ is the partition coefficient of 4-nitrophenol. Full kinetic analysis is described by the reaction rate of each step and the Langmuir adsorption constants of 4-nitrophenol, borohydride and the stable intermediate. We determined the rate constants for both steps by solving the coupled rate equations using the numeric method previously described and fitting the experimental data (average of three experimental trials) [21].

### 2.7. Sequential Reactions

As a measure of nanoreactor stability [12,13], we tested the ability to perform multiple, sequential reactions. Following the standard reaction conditions, sequential additions of 4-nitrophenol (20 µL, 0.01 M) were carried out after the initial addition of 4-nitrophenol and sodium borohydride (333 µL, 6 M) with 3 min between each subsequent addition. No additional sodium borohydride was added. UV-vis absorbance was analyzed continuously over the 35 min time period in accordance with the previously described method.

Bubbles were manually popped prior to each addition of 4-nitrophenol while carefully avoiding contact with the reaction mixture. Due to the presence of bubbles-in-bubbles in subsequent reactions, the start of subsequent reactions was determined by taking an average of 5-consecutive data points after the maximum post-4NP addition absorbance. The last time point with a value equal to, or greater than, this average was defined as the beginning of the reaction. After determining the start of the reaction, the rest of the kinetic analysis follows the procedure described in the Supporting Information. The apparent rate constant of each sequential reaction was determined from the average of three trials.

## 3. Results

### 3.1. Nanoreactor Formulation and Characterization

To incorporate gold nanoparticle catalysts into hierarchical polymer nanostructures, we used polymer directed self-assembly driven by non-covalent, hydrophobic interactions. Specifically, we used flash nanoprecipitation. Flash nanoprecipitation is a rapid, scalable platform for polymer-directed self-assembly of colloidal nanoparticles with versatile materials selection [16,22,23]; therefore, we aimed to examine the effect of various co-precipitants on self-assembly and catalytic performance.

To perform flash nanoprecipitation using castor oil as an initial co-precipitant, hydrophobic gold nanoparticles were dispersed with a dissolved amphiphilic block copolymer and castor oil co-precipitant in a water miscible organic solvent (tetrahydrofuran, THF). This solvent stream was rapidly mixed against water using a confined impinging jet mixer. Upon mixing, the rapid decrease in solvent quality caused the nanoparticles to aggregate, castor oil to precipitate and the block copolymer to micellize directing formation of the hierarchical polymer nanoparticle. This hybrid particle assembly ended when adsorption of the hydrophobic block of the block copolymer prevented further growth and colloidal aggregation. The self-assembled structure was sterically stabilized in water by the hydrophilic block of the block copolymer. Given the molecular weight of the block copolymer, dynamic exchange of the block copolymer did not occur, and the resulting structure was kinetically trapped [2,17,24]. Successful polymer nanoreactor assembly resulted in homogenous dispersions with no macroscopic precipitation of the hydrophobic components, including the gold nanoparticles.

**Figure 1:**
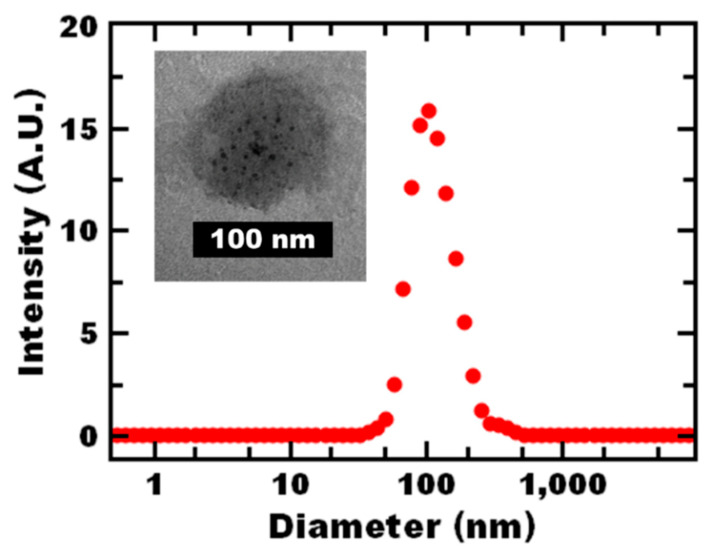
Representative dynamic light scattering (DLS) spectra with a single intensity peak indicative of a uniform castor oil nanoreactor dispersion with representative TEM image as an inset. Gold nanoparticles appear to be incorporated in the nanoreactor; the size is consistent with DLS measurements.

Further evidence of successful self-assembly of dispersed polymer systems was evident by dynamic light scattering (DLS). Specifically, DLS results confirmed that the composite nanoparticles were uniform with a single peak in the size intensity distribution measured by DLS indicating the hydrodynamic diameter was ~100 nm; representative data are shown in red in Figure 1. The UV-vis absorbance of the nanoreactor dispersion was 526 nm, which is comparable to other self-assembled gold–polymer systems and has been attributed to the hydrophobicity of the surrounding environment and the close proximity of the incorporated gold nanoparticles [16]. TEM of the nanoreactors is shown as an inset (Figure 1), with a low magnification TEM image shown in Figure S2 in Supplementary Materials. The nanoreactors appear spherical and the size seen in the images is comparable hydrodynamic diameters reported by DLS. Gold nanoparticles appear to be incorporated in the nanoreactor with multiple gold nanoparticles per nanoreactor. This result is consistent with previous reports [16].

Since flash nanoprecipitation is a versatile platform in terms of materials selection, we prepared a number of other nanoreactor systems with various hydrophobic co-precipitants. We examined dodecane (DD), dodecylamine (DDA) and dodecanethiol (DDT). In the cases of dodecylamine and dodecanethiol, gold-core material interactions may affect both nanoreactor self-assembly as well as catalytic performance. For self-assembling systems that only involved non-covalent, hydrophobic interactions, we studied dodecane and polystyrene (*M*_w_ 750 g/mol PS 750) in addition to castor oil. Using these various hydrophobic co-precipitants, nanoreactors with comparable hydrodynamic size and gold loading were achieved using the constant core volume approach [16]. Specifically, since the densities of the core materials are comparable, using the same co-precipitant concentration in the organic stream during flash nanoprecipitation (11-mg/mL co-precipitant with 1-mg/mL gold nanoparticles and 12-mg/mL block copolymer stabilizer), the hydrodynamic size of the nanoreactors were comparable (Table 1) [16]. As shown in Table 1, all systems formed nanoreactors around 130 nm with low polydispersity (<0.3 PDI) as measured by DLS. The nanoreactor size was stable for at least one week at room temperature, except for the dodecane system (which was not further analyzed).

We also compared the various nanoreactor systems in terms of the amount of gold successfully incorporated into the nanoreactor during self-assembly, which we quantify with the incorporation efficiency (IE). To remove any hydrophobic gold nanoparticles in the dispersion that were not incorporated into the nanoreactors, we performed extraction with ether to selectively remove unincorporated gold nanoparticles while preventing disruption of the nanoreactors (as the PEG stabilizing the nanoreactors was insoluble). The incorporation efficiency for gold nanoparticles via flash nanoprecipitation was greater than 77% ± 5% for all systems compared to 5% for dodecanethiol-capped gold nanoparticles in water (Table S1 in Supplementary Materials). Co-precipitates that interact with gold had the highest incorporation efficiencies. Both dodecanethiol and dodecylamine had incorporation efficiencies greater than 95% (Table S2 in Supplementary Materials). Performing DLS analysis after extraction, we confirmed that the nanoreactors remain intact through the extraction process which we attribute to the insolubility of the amphiphilic polymer, PS–*b*–PEG, in diethyl ether. These results demonstrate that use of co-precipitants with relatively strong interactions with gold (e.g., thiols and amines) increase incorporation efficiency of the gold nanoparticles into the hybrid polymer nanoparticles compared to assemblies that rely solely on noncovalent interactions.

### 3.2. Analysis of Apparent Reaction Kinetics

We began by assessing the catalytic performance of the castor oil nanoreactors using the reduction of 4-nitrophenol by sodium borohydride as a model reaction. Because this reaction proceeds at room temperature without side products and byproducts, it is a well-established model reaction for kinetic studies using gold nanoparticles [16,18,20,21,25,26,27]. We also note that reduction of 4-nitrophenol is important in waste water treatment [28,29] and the product resulting from the model reaction, 4-aminophenol, has several applications, e.g., a corrosion inhibitor as well as an intermediate in the pharmaceutical industry [30]. A schematic of the nanoreactor structure and these potential catalytic applications are illustrated in Figure S3 in Supplementary Materials. We confirmed that the nanoreactors were stable following reaction. Some minor swelling was observed; the size increased from 113 ± 10 (PDI 0.184 ± 0.011) before reaction to 154 ± 8 (PDI 0.220 ± 0.012) after reaction.

**Figure 2:**
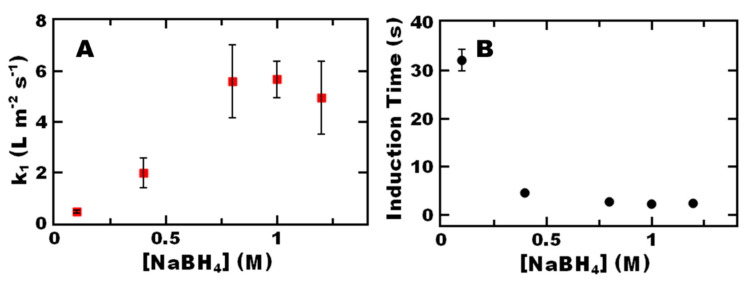
Effect of varying concentration of sodium borohydride concentration used in the 4-nitrophenol reduction reaction on (**A**) reaction rate constant and (**B**) induction time using castor oil nanoreactors (CO NRs). The initial concentration of 4-nitrophenol in each reaction was 0.1-mM. A) A plateau in apparent reaction rate constant per surface area of gold (*k*_1_) is observed for CO NR above 0.8-M NaBH_4_ concentration. CO NR show a maximum *k*_1_ of 5.7 ± 0.7 L m^−2^ s^−1^. B) The CO NR system shows a maximum induction time of 32 ± 2 s.

To facilitate pseudo-first order rate kinetics where the reaction rate is independent of sodium borohydride concentration, we first examined the effect of sodium borohydride concentration on reaction kinetics [20,21]. For the CO NRs prepared via flash nanoprecipitation, we observe the apparent reaction rate per surface area of gold (*k*_1_) increased with sodium borohydride concentration until it plateaus at a sodium borohydride concentration of 1.0 M sodium borohydride (Figure 2A). Thus, the bulk sodium borohydride concentration in the reaction medium was 1.0 M to achieve pseudo-first order rate kinetics i.e., reaction is independent of sodium borohydride concertation. These results are similar to previous reports for gold nanoparticle-polymer nanoparticle systems [21]. This plateau has been attributed to active site blocking predicted by Langmuir-Hinshelwood kinetics when one reagent predominantly occupies the catalyst surface [21,31,32]. Interestingly, the sodium borohydride concentration required to achieve a plateau for the nanoreactors was a 10-fold increase from previous reports [21,32] which could suggest differences in localized reagent concentrations compared to other systems, i.e., a difference between the bulk sodium borohydride concentration and the concentration at the catalyst surface due to partitioning and diffusion.

We also note that increasing the concentration of sodium borohydride decreases the induction time (Figure 2B). These results are consistent with the trends in which changes in induction time and reaction kinetics were inversely proportional to one another reported by Ballauff [21]. The change in induction time that we observe in this case appears to be related to an increase in sodium borohydride concentration which has recently been attributed to consumption of oxygen by sodium borohydride [33].

To further understand the potential effect of hierarchical structure of the self-assembled, amphiphilic particles achieved during flash nanoprecipitation on catalytic performance, we compared the catalytic performance of the castor oil nanoreactors to gold nanoparticles added to preformed castor oil nanoparticles. We further compared the results with gold nanoparticles with hydrophilic stabilizers (PEG-coated gold nanoparticles and citrate-stabilized gold) at equivalent gold concentrations measured at 1.0-M NaBH_4,_ 0.1-mM 4-nitrophenol. Thus, castor oil nanoparticles of comparable hydrodynamic size were formulated (Table S2). Following flash nanoprecipitation, hydrophobic gold nanoparticles dispersed in THF were added to the dispersed polymer nanoparticles. No macroscopic aggregation of the gold nanoparticles in the aqueous phase was observed. Rather, the gold nanoparticles associated with the polymer nanoparticle via nonspecific adsorption (presumably with the PEG surface). In this study, we were interested in comparing the catalytic performance of the castor oil nanoreactors to gold nanoparticles added to preformed castor oil nanoparticles to PEG-coated gold nanoparticles and citrate-stabilized gold nanoparticles (hydrophilic stabilizers) to determine the effect of the hierarchical, amphiphilic nanoreactor structure achieved by flash nanoprecipitation.

Interestingly, we observed that the reaction rate normalized per surface area per gold (*k*_1_) for the gold nanoparticles added to the preformed gold nanoparticles were comparable to the PEG-coated gold nanoparticles and citrate-stabilized gold nanoparticles (Table 2). Notably, the *k*_1_ of the castor oil nanoreactors was over 2.7-fold faster than these other systems. These results suggest that the hierarchical, amphiphilic structure of the nanoreactors achieved by self-assembly with the amphiphilic polymer, castor oil and gold nanoparticles is beneficial for catalytic performance compared to gold nanoparticles with hydrophilic stabilizers.

### 3.3. Analysis of Intrinsic Reaction Kinetics via Partition Coefficient Analysis and Langmuir–Hinshelwood Kinetics

Rate acceleration in self-assembled amphiphilic systems has been previously observed and attributed to increased local concentrations of the reactants [34]. To quantify this difference in concentration in this system, we measured the partition coefficient of 4-nitrophenol between water and castor oil. The castor oil:water partition coefficient was 7.81 ± 0.16 indicating an increased local concentration within the nanoreactor was possible. We used this information to next examine the intrinsic kinetics of the 4-nitrophenol reduction on the gold nanoparticle catalyst surface based on the Langmuir–Hinshelwood mechanism [21,35]. For nanoreactors, the concentration in 4-nitrophenol was calculated based on the bulk concentration and the experimentally determined core material:water partition coefficient. For gold nanoparticles added to preformed polymer nanoparticles, the 4-nitrophenol concentration was taken to be the concentration in the bulk aqueous phase. Initially, we used a two-step reaction model involving reduction of 4-nitrophenol to 4-hydroxylaminophenol and the rate limiting step of 4-hydroxylaminophenol to 4-aminophenol previously used for gold-polymer systems [21,32]. The two-step reaction is evident by a change in reaction rate typically when the conversion of 4-nitrophenol is above 30% [21]. While we observed a change in reaction rate, it generally occurred around 70% conversion of 4-nitrophenol. Fitting data up until 70% conversion resulted in non-real solutions. Poor fits and large error (greater than 100%) were observed when fitting data up to 30%.

Therefore, we used an alternative model. Specifically, the data up to 30% conversion was modeled using a single reaction step and no observed change in reaction rate based on previous reports of polymer–gold systems [18], according to:

(4) $$-\left(\frac{dc_{nip}}{dt}\right)=k_{0}S\frac{{\left(K_{nip}P_{nip}c_{nip}\right)}^{n}\left(K_{BH_{4}}c_{BH_{4}}\right)}{{\left[1+{\left(K_{nip}P_{nip}c_{nip}\right)}^{n}+K_{BH_{4}}c_{BH_{4}}\right]}^{2}}$$

where *c*_nip_ is the bulk aqueous phase concentration of 4-nitrophenol, *k_0_* is the reaction rate constant of the single step reaction, *S* is the reaction solution specific catalyst surface area concentration, *P*_nip_ is the core material:water partition coefficient of 4-nitrophenol, *n* is the reaction order and *K*_nip_ and *K_BH4_* are the Langmuir–Hinshelwood adsorption constants of 4-nitrophenol and sodium borohydride, respectively.

Using the adsorption parameters previously reported [21], the calculated rate constants are reported in Table 3. Notably, the rate constants for castor oil nanoreactors and gold nanoparticles added to preformed castor oil nanoparticles were comparable. Further, the measured intrinsic kinetic parameters were comparable to PEG-coated gold nanoparticles and citrate-stabilized gold nanoparticles. These results suggest that the intrinsic kinetics of the catalysts for all the systems are comparable once the partition coefficient was used to estimate the localized 4-nitrophenol concentration. Thus, incorporating the gold nanoparticles into the nanoreactors via self-assembly does not adversely impact the intrinsic catalytic performance compared to adding gold nanoparticles to preformed castor oil nanoparticles or citrate-stabilized gold nanoparticles. Therefore, the difference in apparent catalytic performance can be attributed to an increase in local reactant concentration.

Comparing the rate constants from the Langmuir–Hinshelwood model to previous literature, we found that the rate constants for the systems prepared by flash nanoprecipitation (Table 3) were at least two orders of magnitude greater than previously reported gold–polymer systems [18]. Their system and their analysis were comparable to the gold nanoparticles added to preformed polymer particles. These results further indicate that flash nanoprecipitation is a promising platform for producing nanoreactors while preserving the intrinsic activity of the catalyst. Furthermore, the catalytic performance can be affected by carefully considering solubility of the reactants when selecting materials for the nanoreactor.

### 3.4. Comparison of Catalytic Performance

Next, we were interested in comparing the apparent catalytic performance of the castor oil nanoreactors to the other nanoreactor systems. We began by comparing the castor oil to other nanoreactor systems including PS 750 nanoparticles without gold (less than 1 ppm by ICP-OES) as a negative control (Table 4). We used 1.0-M sodium borohydride concentration and 0.1-mM 4-nitrophenol concentration to achieve pseudo-first-order rate kinetics. Interacting core materials such as dodecanethiol and dodecylamine did not catalyze the 4-nitrophenol reduction, likely due to active site blocking. While gold-core material interactions were beneficial for improving incorporation efficiency, there was a trade off with catalytic performance. Therefore, nanoreactors prepared via flash nanoprecipitation, which can be driven by hydrophobic interactions, may offer advantages over self-assembled systems that rely on gold–polymer interactions [18,31].

Notably, at these reaction conditions the apparent reaction rate constant per surface area of gold (*k*_1_) for both PS 750 NR and CO NR were at least 2-fold greater than ligand-free gold nanoparticles [32] and approximately 10-fold greater than gold nanoparticles functionalized with pH responsive poly(acrylic acid) [26].

For the non-interacting core materials, the apparent reaction rate for CO NRs was over 8-fold greater than PS 750 NRs with apparent reaction rate constant per surface area of gold of 5.7 ± 0.7 L m^−2^ s^−1^ for CO NRs compared to 0.7 ± 0.1 L m^−2^ s^−1^ for PS 750 NRs (Table 4). We further investigated the difference between the PS 750 and CO nanoreactor systems. Based on the apparent kinetics, we considered potential mass transfer limitations using scaling analysis and the second Damkohler number (*DaII*), i.e., a ratio of the reaction and diffusion rates given by,

(5) $$DaII=\frac{k_{app}C^{n-1}}{\beta a}$$

where *n* is the reaction order, *β* is the mass transport coefficient (which is a quotient of the diffusion coefficient and the characteristic length of the system) and *a* is the interfacial area. Using a previously established NMR method [16], the diffusion coefficient for 4-nitrophenol in CO NR was determined to be 1.70 ± 0.02 × 10^−8^ m^2^/s (Figure S1 in Supplementary Materials) which is slightly lower than for polystyrene 1.91 ± 0.01 × 10^−8^ m^2^/s [16]. Based on these experimentally determined diffusion coefficients, the second Damkohler number was found for all systems to be on the order of 10^−4^ or smaller, signifying there were no diffusion limitations for either the CO NRs or PS 750 NRs.

Based on our results, we posited that the difference in apparent catalytic performance may be attributed to differences in local reactant concentration and specifically 4-nitrophenol concentration. Hanson solubility parameters suggest that castor oil is a better solvent for 4-nitrophenol than polystyrene (Table S3 in Supplementary Materials) and the effective 4-nitrophenol concentration within CO NRs would be higher than PS 750 NRs. In order to confirm this difference in solubility of 4-nitrophenol in different nanoreactor microenvironments, we measured the partition coefficient of 4-nitrophenol between water and castor oil or toluene (to mimic PS). As seen in Table 5, the core material:water partition coefficient for castor oil was 7.81 ± 0.16 compared to 0.09 ± 0.01 for toluene. The higher core material:water partition coefficient for castor oil compared to toluene suggests that the effective concentration of 4-nitrophenol in the CO NRs would be higher than a PS NR contributing to enhanced apparent catalytic performance. Taken together, these results indicate that 8-fold enhancement in catalytic activity with the castor oil nanoreactors compared to the PS 750 nanoreactors could be attributed to the increased solubility of the reactants rather than changes in the intrinsic catalytic properties or differences in mass transfer. Broadly, ability to enhance the apparent catalytic performance through selection of the core material (i.e., choosing a core material that the reactant is highly soluble in) is promising approach for rational design of nanoreactors. Thus, we note Hansen solubility parameter distance (RA^2^) may be a useful tool for future nanoreactor design.

In addition to comparing the PS 750 and CO nanoreactor systems to each other, comparisons to previously reported nanoparticle-based catalyst systems were also made. The turnover frequency (TOF) is related to apparent reaction rate constant, the initial amount of 4-nitrophenol and the amount of gold catalyst and is a useful metric for comparing across various catalytic systems [36]. The turnover frequency (TOF) was calculated according to:

(6) $$TOF=\frac{k*n_{o_{4NP}}}{n_{Au}}$$

where *k* is the apparent reaction rate constant, while *n_Au_* and *n_0,AU_* are the molar amounts of gold and initial 4-nitrophenol in the reaction, respectively [36]. Based on the observed reaction rate constants, the TOFs for CO and PS 750 NRs were approximately 300,000 and 30,000 min^−1^, respectively (Table 6). These TOFs were higher than typically reported for gold-based catalysts (~1–2 min^−1^) [37,38,39]. The TOF of the self-assembled nanoreactors were also higher than gold–polymer systems previous reported such as gold nanoparticles coated on poly(glycidyl methacrylate) microspheres modified with poly(allylamine hydrochloride) (PAH) and negatively charged preformed gold nanoparticles (AuNPs) with a TOF ~15,000 min^−1^ [40], as well as dendrimer encapsulated gold nanoparticle catalysts TOF ~2,000 min^−1^ [41], as well as gold nanoparticles on N-containing polymer nanospheres TOF ~17,000 min^−1^ [42]. These results suggest that self-assembled nanoreactors produced via flash nanoprecipitation have promising catalytic performance. Furthermore, these results indicate the catalytic performance is highly influenced by the self-assembled polymer microenvironment.

### 3.5. Nanoreactor Reuse

Finally, we consider the ability to use the nanoreactors for multiple reactions as an initial step to understanding the robustness of the castor oil nanoreactors. Importantly, full conversion of the 4-nitrophenol was achieved for the CO nanoreactor systems within 3 min using 1-M NaBH_4_ for at least 10 sequential reactions. This indicates that the nanoparticle catalysts incorporated in the kinetically trapped polymer nanoreactor systems resist irreversible aggregation that would result in a complete loss of activity [27].

**Figure 3:**
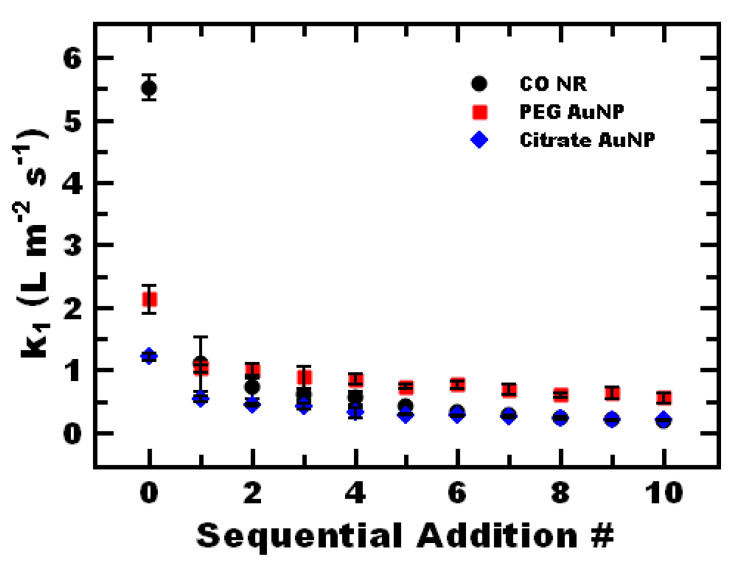
Sequential additions of 4-nitrophenol performed with PEG-stabilized gold nanoparticles (PEG AuNP) (red), CO NR (black), compared to citrate-stabilized gold nanoparticles (citrate AuNP) (blue) with 1-M NaBH_4_.

When performing sequential reactions, a decrease in activity was seen when sequentially adding 4-nitrophenol into the reaction mixture under 1-M NaBH_4_ conditions (Figure 3). Generally, the greatest decrease in activity occurred after the first reaction, with less significant changes with additional reactions. For example, CO NR undergo an immediate 5-fold decrease in activity from the initial 4-nitrophenol reduction to the first sequential reaction. Following the first reaction, the tenth sequential reaction is 6-fold slower than the first sequential reaction. Overall, the tenth reaction is 30-fold slower than the initial reaction, with a final minimum activity of 0.18 ± 0.01 L m^−2^ s^−1^. Similarly, the largest decrease in reaction rate for the citrate-stabilized and PEG-coated nanoparticles occurred after the first reaction (Figure 3). Interestingly, the CO NR and citrate-stabilized gold nanoparticles plateaued to the same value following the first reaction. This result indicated that the decrease in rate was likely inherent to the gold nanoparticle. Decreases in reaction rate constant with multiple reactions has been previously observed and attributed to partial gold nanoparticle aggregation in the presence of sodium borohydride [26,27] and/or competitive adsorption on the catalyst surface [46].

We note that that the PEG-stabilized gold nanoparticles better retained their activity with sequential reactions compared to the other systems. The CO NR had a greater initial activity than PEG-stabilized, 5.7 ± 0.7 L m^−2^ s^−1^ compared to 2.1 ± 0.2 L m^−2^ s^−1^_,_ respectively. After 10 sequential additions the PEG AuNP had 3-fold greater activity than the CO NR (0.56 ± 0.8 and 0.18 ± 0.01 L m^−2^ s^−1^_,_ respectively). These results may indicate a trade off between stability and initial apparent kinetics with hydrophilic stabilizers utilizing gold–thiol interactions for self-assembly and the amphiphilic nanoreactors produced here via polymer directed self-assembly leveraging non-covalent interactions.

Notably, even after 10 sequential reactions, the remaining catalytic activity of the castor oil nanoreactors exceeds that observed in previously reported gold nanoparticle systems. For example, the apparent reaction rate constant *k*_1_ of CO NR after 10 sequential additions was 3-orders of magnitude greater than previously reported gold nanoparticle–polymer brushes systems [47] and roughly equivalent to ligand free gold nanoparticles [32]. Further, after 10 sequential additions (despite the 3-fold decrease in activity), the TOF was 23,000 min^−1^ which was higher than typically reported for gold-based catalysts (~1–2 min^−1^) [37,38,39] and comparable to previously reported gold–polymer based systems [40,42].

## 4. Conclusions

Using flash nanoprecipitation, we leveraged non-covalent interactions for polymer directed self assembly of amphiphilic nanoreactors incorporating gold nanoparticle catalysts. We examined the effect of co-precipitant on self-assembly and catalytic performance. We determined that core materials that interact with gold are beneficial for improving incorporation efficiency of the gold nanoparticles into the nanocomposite nanoreactor during self-assembly but decreased catalytic performance. Catalytic performance was strongly affected by the co-precipitant that formed the hydrophobic microenvironment for reaction. For example, the apparent reaction rate per surface area using castor oil (CO) was over eight-fold greater than polystyrene (750 g/mol, PS 750). Examining the differences in partition coefficient, the increase in apparent catalytic performance could be attributed to differences in reactant solubility rather than differences in mass transfer or intrinsic kinetics. Overall, higher reactant solubility enhances apparent reaction rates and may be a useful design parameter for material selection in future nanoreactor studies.

## Figures and Tables

**Table 1:** Size and stability of various nanoreactor prepared via flash nanoprecipitation.

System	Size (nm)	PDI	Stability *
CO NR	113 ± 10	0.184 ± 0.011	Yes
PS 750 NR	124 ± 6	0.131 ± 0.012	Yes
Dodecane NR	168 ± 6	0.108 ± 0.003	No
Dodecylamine NR	143 ± 23	0.263 ± 0.019	Yes
Dodecanethiol NR	101 ± 6	0.087 ± 0.028	Yes

* Stability defined as change in diameter < 30% and a PDI < 0.3 after 1 week of storage at room temperature as measured by DLS.

**Table 2:** Reaction kinetics of castor oil nanoreactors (CO NR) compared to gold nanoparticles added to preformed castor oil nanoparticles (CO NP w AuNP), PEG-coated gold nanoparticles (PEG-coated AuNP) and citrate-stabilized gold nanoparticles (citrate-stabilized AuNP) at equivalent gold concentrations measured at 1.0-M NaBH_4_ and 0.1-mM 4-nitrophenol.

Nanoreactor	*k*_1_ (L m^−2^ s^−1^)
CO NR	5.7 ± 0.7
CO NP w AuNP	1.6 ± 0.4
PEG-coated AuNP	2.1 ± 0.2
Citrate-stabilized AuNP	1.2 ± 0.1

**Table 3:** Langmuir–Hinshelwood single-step fitted kinetic parameters.

System	*k*_0_ (mol m^−2^ s^−1^)
CO NR	0.0103 ± 0.0015
CO NP w AuNP	0.0084 ± 0.0001
PEG AuNP	0.0151 ± 0.0008
Citrate AuNP	0.0100 ± 0.0005
Gold nanoparticles coated on polystyrene microspheres grafted with poly([2-aminoethyl]-methacrylate hydrochloride) from ref [18]	2.27 ± 0.34 × 10^−4^

**Table 4:** Reaction kinetics of different polymer nanoreactors at 1.0-M NaBH_4,_ 0.1-mM 4-nitrophenol.

Nanoreactor	*k*_1_ (L m^−2^ s^−1^)
CO NR	5.7 ± 0.7
PS 750 NR	0.7 ± 0.1
Dodecylamine NR	<0.001 ± 0.001
Dodecanethiol NR	<0.001 ± 0.001
PS 750 NP without AuNP	0.001 ± 0.001

**Table 5:** Core material:water partition coefficients for 4-nitrophenol (4NP).

Core Material Organic Phase	Core Material: Water Partition Coefficient of 4NP
Castor Oil	7.81 ± 0.16
Toluene	0.09 ± 0.01

**Table 6:** Turnover frequencies (TOF) of nanoreactors compared to other hybrid nanoparticle systems.

System	TOF (min^−1^)	Reference
CO NR	29.7 ± 3.8 × 10^4^	This Paper
PS 750 NR	3.3 ± 0.2 × 10^4^	This Paper
Gold nanoparticles (NP) coated on poly(glycidyl methacrylate) microspheres with poly(allylamine hydrochloride)	1.5 × 10^4^	[40]
Gold NPs stabilized by amphiphilic dendronized diblock copolymer (hydrophilic triethylene glycol (TEG) and hydrophobic ferrocenyl (Fc) groups)	440	[43]
Gold NPs stabilized by polyamidoamine dendrimer	2 × 10^3^	[41]
Gold NPs stabilized by PEI derivatives	1.5 × 10^4^	[37]
PVP-stabilized gold NPs	0.8	[44]
Citrate-stabilized gold NPs	3.0	[45]

## Supplementary Materials

The following are available online at https://www.mdpi.com/2073-4360/12/8/1774/s1, Figure S1: Diffusion Coefficient of 4NP in CO NR, Figure S2: Low magnification TEM of castor oil nanoreactors showing multiple nanoreactors, Figure S3: Schematic of the nanoreactor structure and their potential application in catalysis. Table S1: Characterization of gold concentration in the nanoreactor dispersion and incorporation efficiency (IE) of the gold nanoparticles into the nanocomposite particles, Table S2: Size of castor oil nanoreactors prepared by flash nanoprecipitation compared to gold nanoparticles added to preformed castor oil nanoparticles. Table S3: Hansen Solubility Parameters of Various Compounds
