# Supplementary material for: Accelerated Reaction Rates within Self-Assembled Polymer Nanoreactors with Tunable Hydrophobic Microenvironments
Source: Polymers (Basel). 2020 Aug 7;12(8):1774. doi: 10.3390/polym12081774 (PMC7463608; doi:10.3390/polym12081774)
Supplement: Supplementary file 1 [file polymers-12-01774-s001.pdf]

Supplementary Information

Accelerated Reaction Rates within Self-Assembled Polymer Nanoreactors with Tunable Hydrophobic Microenvironments

Andrew Harrison , Michael P. Zeevi, Christopher L. Vasey, Matthew D. Nguyen and Christina Tang ,*

Virginia Commonwealth University, College of Chemical and Life Sciences Engineering, Richmond, VA 23284-3028, USA; harrisona3@vcu.edu (A.H.); zeevimp@vcu.edu (M.P.Z.); vaseycl@vcu.edu (C.L.V.) nguyenmd4@vcu.edu (M.D.N.)

* Correspondence: ctang2@vcu.edu

S1. Methods

S1.1.1: Kinetic Analysis

Generally, gold nanoreactors (NR) (120 uL, 0.0079 mol% AuNP) produced using FNP were diluted with water (1.527 mL) in a quartz cuvette with a stir bar. The reaction was monitored under stirring with an Ocean Optics FLAME-S-VIS-NIR-ES using an HL-2000-FHSA light source (300–1200 nm), with a CUV-UV cuvette holder (Ocean Optics) placed on a stir plate. The spectrometer was blanked to the reaction mixture. Typically, 4-nitrophenol (20 uL of 0.01M solution) was added to the reaction mixture, and data collection began. After 1 minute, sodium borohydride (333 uL of a 6-M solution) was added to the stirring reaction mixture. Scans were taken every millisecond and averaged over 10 scans with data recorded every 50 milliseconds. The reaction was tracked by monitoring the change in intensity of 4-nitrophenol peak at 425 nm. Absorbance at 425 nm was background corrected be subtracting out the time dependent 600 nm absorbance intensity from the corresponding data point. The induction period was fit to a line, and the data set was normalized to the maximum data point among the first witnessed domain of, at least, five consecutive points to fall 1% below the fitted line. Similar to previous studies [1], the induction time and apparent reaction rate were determined from the absorbance as a function of time.

S1.1.2: Induction Time Determination

Initially, the spectra of the polymer nanoreactor dispersion was recorded as a reference to be subtracted from subsequent spectra as background. The 4-nitrophenol was added resulting in an increase in absorbance at 425 nm. The increase in absorbance due to the addition of 4-nitrophenol occurs within 30 seconds. After 1 minute, the sodium borohydride is added, which results in a further increase in absorbance at 425 nm due to formation of the 4-nitrophenolate ion. The initiation of reaction conditions corresponding to the start of the induction period was defined as the time at which the absorbance at 425 nm increased to at least 10% of the absorbance at 45 seconds. The induction time itself is characterized by a slow decrease (0.002 Abs/s) in absorbance that is followed by a sharp (> 0.01 Abs/s) decline in absorbance indicating beginning of the reduction reaction, which signifies the end of the induction period. Changes in the slope of the absorbance vs. time was used to quantitatively determine the induction time. Specifically, the absorbance vs. time during the induction time was presumed to be adequately described by a line calculated from fitting the initial region of the induction period (no less than 10% of the total approximate

induction time domain). For example, if the induction period lasted roughly 300 seconds based upon visual inspection, the linear fit of the induction time was based on the first 60 seconds of the induction time. Next, the maximum value within the first five or more, consecutive data points which were at least 1% less than the fitted line was used to normalize the data set. Importantly, these consecutive data points must occur after the start of the induction time period. The time point designating the end of the induction time was then defined as the last occurrence of the normalized data point with a value of 1.

S1.1.3: Apparent Reaction Rate Constant

For analysis of the reaction rate, the natural log of the normalized absorbance was plotted with respect to time and regions of two distinct slopes were observed. The first region has been attributed to formation of an intermediate [2]. In order to avoid analysis of the intermediate reaction, the apparent reaction rate was calculated from the second region corresponding to when the normalized absorbance fell below 0.67. Data corresponding to a 15% conversion were analyzed when determining apparent reaction rate. If the 15% conversion domain contained less than 4 data points, one data point above the determined region and below the region was included, such that no analysis included less than three data points. The induction time and apparent reaction rate are reported as the average ± standard deviation of three experimental trials.

With a sufficiently large excess of sodium borohydride compared to 4-nitrophenol, the reaction kinetics can be described by pseudo-first-order kinetics. For heterogeneous catalysts, the apparent rate constant is assumed to be proportional to the surface of the catalyst described by [2]:

$$-\frac{dc}{dt} = k_{app}c = k_1 Sc \quad \text{(S1)}$$

where c is the concentration of 4-nitrophenol at time (t), k_{app} is the apparent rate constant, k_1 is the rate constant normalized to surface area of gold nanoparticles per unit volume of the reaction. Experimentally, k_1 is determined by the change in 4-nitrophenol concentration after the induction period and the mass of gold measured by ICP (surface area calculated assuming 5 nm spherical particles). Pseudo-first-order rate kinetics are characteristically described by a linear fit when the natural log of the reagent concentration is plotted with time.

S1.1.4: NMR Measurements

To evaluate effective transport of the 4-nitrophenol, ^{1}H-NMR spectroscopy and pulsed field gradient (PFG) NMR, combined with saturated transfer difference (STD) spectroscopy, using a Bruker 800 MHz cryo-probe (Billerica, MA, USA) was performed in accordance with the methods described previously (our paper). Briefly, 4-nitrophenol molecules in close proximity to the nanoreactor core were analyzed based on spin diffusion of selectively saturated core material (polystyrene or castor oil), in conjunction with an applied magnetic field gradient. Relevant peak intensities were analyzed as a function of gradient strength to determine the diffusion coefficient of 4-nitrophenol molecules of interest. Since nanoreactors diffuse in free solution at least 3 orders of magnitude slower than molecules, the measured diffusion coefficient was considered the effective diffusion coefficient of the solute within the nanoreactor [3,4]. A detailed method explanation has been published previously [26].

S2.7: Diffusion Coefficient of 4-Nitrophenol in CO NR

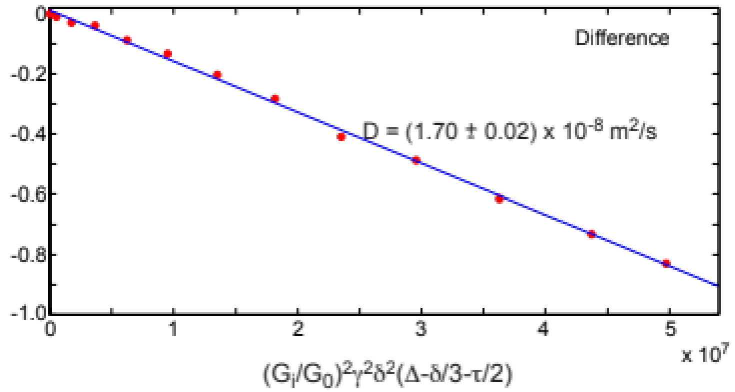

Figure S1: Results of saturation-transfer-differentiated PFG-NMR of 4-nitrophenol in a solution of castor oil nanoreactors. The blue line corresponds to the curve fit. The difference spectra corresponds to signal from the 4-nitrophenol in closest proximity to the hydrophobic phase (within the nanoreactors) which we interpret as an effective diffusion coefficient of 4-nitrophenol within the nanoreactors of 1.7 x 10^{-8} m^2/s.

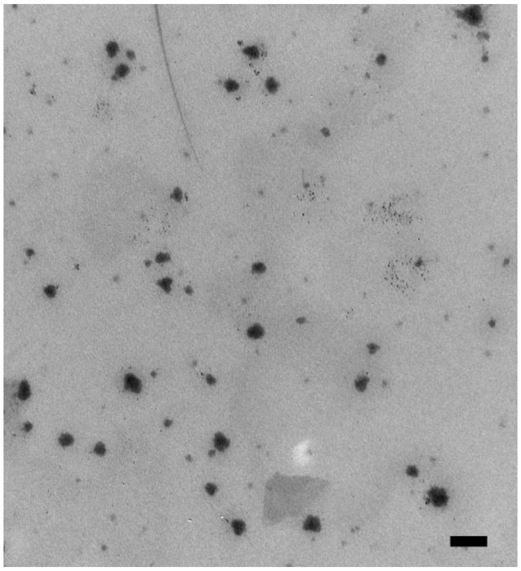

Figure S2. Low-magnification TEM of castor oil nanoreactors showing multiple nanoreactors. The scale bar represents 100 nm.

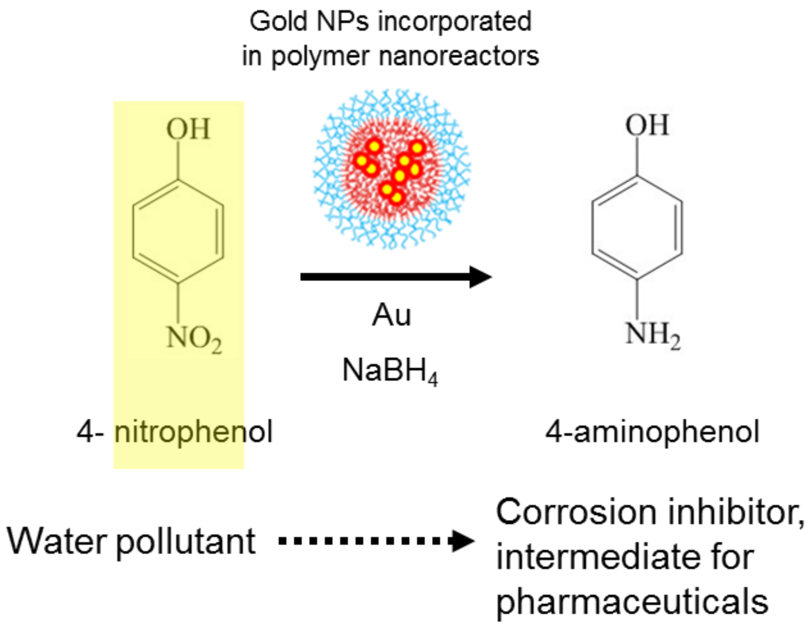

Figure S3. Schematic of the nanoreactor structure and their potential application in catalysis.

Table S1: Characterization of gold concentration in the nanoreactor dispersion and incorporation efficiency (IE) of the gold nanoparticles into the nanocomposite particles.

System	[Au] (µg/mL)	IE (%)
PS 750 NR	137 ± 1	85 ± 2
PS 750 NP w AuNP	119 ± 6	89 ± 7
CO NR	106 ± 1	84 ± 3
CO NP w AuNP	118 ± 11	94 ± 2
Dodecylamine NR	85 ± 1	98 ± 3
Dodecanethiol NR	101 ± 3	97 ± 1
Hydrophobic AuNP in water	71 ± 1	5 ± 1

Table S2: Size of castor oil nanoreactors prepared by flash nanoprecipitation compared to gold nanoparticles added to preformed castor oil nanoparticles.

System	Size (nm)	PDI
CO NR	113 ± 10	0.184 ± 0.011
CO NP w AuNP	136 ± 7	0.147 ± 0.006

Table S3: Hansen solubility parameters of various compounds

Compound	δ_d ($MPa^{1/2}$)	δ_p ($MPa^{1/2}$)	δ_h ($MPa^{1/2}$)	4NP RA^2
Polystyrene [9]	18.5	4.5	2.9	244
Toluene [10]	18	1.4	2.0	336
Castor Oil [11]	15.9	4.6	12	170
Ethanol [10]	15.8	9.8	19.4	120
PEO 4000 [10]	21.5	10.9	13.1	13
Methacrylamide [10]	15.8	12.1	12.8	64
PMMA 30 [10]	17.2	7.2	3.5	120
4-nitrophenol [10]	20	14.5	14.2	0

The HSP distance or RA^2, is a measure of how alike two compounds are. The smaller the RA^2 value, the more likely they are to be compatible with each other. The HSP distance is calculated as follows:

$$RA^2 = 4\left(\delta_{d_1} - \delta_{d_2}\right)^2 + \left(\delta_{p_1} - \delta_{p}\right)^2 + \left(\delta_{h_1} - \delta_{h_2}\right)^2 \quad \text{(S2)}$$

Of the hydrophobic core materials chosen, castor oil presents the lowest RA^2 value and would likely interact with 4-nitrophenol the most favorably.
